# Comprehensive Analysis of Tumor Immune Microenvironment Characteristics for the Prognostic Prediction and Immunotherapy of Oral Squamous Cell Carcinoma

**DOI:** 10.3389/fgene.2022.788580

**Published:** 2022-04-08

**Authors:** Yijie Zhao, Dongyi Chen, Junhao Yin, Jian Xie, Chun-yu Sun, Mengmeng Lu

**Affiliations:** ^1^ Department of Oral and Maxillofacial Surgery, Shanghai Stomatological Hospital, Fudan University, Shanghai, China; ^2^ Department of Prosthodontics, School and Hospital of Stomatology, Tongji University, Shanghai Engineering Research Center of Tooth Restoration and Regeneration, Shanghai, China; ^3^ Department of Oral Surgery, Shanghai Ninth People’s Hospital, College of Stomatology, Shanghai Jiao Tong University School of Medicine, Shanghai, China; ^4^ Department of Urology, Huashan Hospital, Fudan University, Shanghai, China

**Keywords:** OSCC, time score, immunotherapy, prognosis, biomarker

## Abstract

**Background:** Oral squamous cell carcinoma (OSCC) is the most common cancer of oral and maxillofacial region. A recent clinical research has shown that tumor immune microenvironment (TIME)cells are closely related to immunotherapy sensitivity and OSCC prognosis. Nonetheless, a comprehensive analysis of TIME in OSCC has not been reported.

**Methods:** Bioinformatics and computational algorithms were employed to determine the significance of TIME cells in 257 OSCC patients. TIME scores were measured by three TIME models, and then used to evaluate the prognosis of OSCC patients.

**Results:** High TIME score was characterized by better prognosis in OSCC patients less than 60 years old, overexpression of immunotherapy targets (e.g., PD-1 and CLTA-4), and higher T-cell activity to inhibit tumor growth. Besides, poor prognosis was associated with low time score.

**Conclusion:** TIME score exhibited potential as a prognostic biomarker and an indicator in predict immunotherapeutic outcomes. Through the understanding of TIME model, this study can provide a better scheme for immunotherapy as the effective treatment of OSCC patients in the future.

## Introduction

Oral squamous cell carcinoma (OSCC) is a malignant cancer derived from oral epithelium, and is the most harmful tumor in the head and neck area (Dourado et al., 2018). OSCC is originated from gingiva, hard palate, tongue, buccal mucosa, lips and other organs, which accounted for more than 350,000 new cancers and 170,000 deaths in 2018 (Bray et al., 2018; Sun et al., 2020). In addition, OSCC is prone to distant metastasis, causes the loss of oral function, affects the quality of life, and ultimately results in a life-threatening condition (Linsen et al., 2018; Lu et al., 2019).

At present, OSCC treatment is mainly surgery-based comprehensive sequence therapy, especially the triple combination therapy of chemotherapy, radiotherapy and surgery (Huang and O Sullivan, 2013; Chai et al., 2020). However, the 5-years overall survival rate of OSCC patients is only about 60% (Chinn and Myers, 2015). Thus, it is imperative to find a more effective treatment for current status. Previous research has shown that immune microenvironment is closely related to tumor progression and prognosis (Binnewies et al., 2018; Bray et al., 2018). Tumor-infiltrating immune cells are involved in the establishment of tumor immune microenvironment, for example, tumor-associated macrophage (TAMs) is involved in the progression and metastasis of OSCC patients (Zhang X et al., 2020; Park et al., 2020). Moreover, IL-4, a cytokine produced by TAMs, promotes tumor angiogenesis, while IL-10 increases tumor cell migration and invasion through Gas6/Axl pathway (Yang W et al., 2020; Tanaka and Siemann, 2020; Wang et al., 2020). Besides, tumor-infiltrating lymphocytes can promote anti-tumor immunity, for example, CD4+T cells have cytotoxicity and their combination with CD8+T cells can exert better inhibitory activity on tumor cells (Hoesli et al., 2018; Lin et al., 2020). As a result, immunotherapy shows a good application prospect for OSCC treatment (Zhu et al., 2019a).

So far, the TIME characteristics of OSCC patients have not been fully elucidated through a comprehensive landscape. Previous immunotherapies for OSCC have only targeted individual genes, however, systematic analysis of immune-related genes is lacking. Therefore, CIBERSORT and ESTIMATE algorithms were used to analyze the gene expression profiles of a large number of OSCC samples and obtain a comprehensive prospect of intratumoral immune status (Wang et al., 2020; Zhao et al., 2020; Peng et al., 2021). TIME scores were then applied to predict OSCC patients’ results and their responses to immunotherapy.

## Materials and Methods

### OSCC Datasets

From the OSCC database (http://www.cancergenome. nih.gov/), TCGA RNA-seq dataset and the respective clinical data of 257 oral squamous cell carcinoma samples (e.g., palate samples, gingival samples, tongue root samples, unspecified oral samples, mouth floor samples, unspecified tongue samples, and others) were downloaded (Zollinger et al., 2018; Chen et al., 2020). The baseline and clinical data, including sex, age, tumor grade, race, survival status, survival time and pathologic stage, were collected. The login numbers of all specimens are listed in Supplementary file S1. We also searched the human expression profile of oral cancer from NCBI GEO (accession number: GSE41613). The public domain names and direct Web links are summarized in Supplementary File S2.

### Consensus Clustering for Tumor Immune Microenvironment Cells

CIBERSORT is a deconvolution method applied for the characterization of cell composition of a complex tissue (Peng et al., 2021). LM22 is a leukocyte gene signature matrix that contains the expression levels of 547 genes for distinguishing 22 human hematopoietic cell phenotypes (Mills et al., 2021). In this study, CIBERSORT combined with LM22 signature matrix were used to quantify the infiltration levels of twenty-two immune cells in OSCC. Subsequently, consensus clustering was performed using the ConsensuClusterPlus R package (Yang et al., 2017). The cumulative distribution function curve of a consensus matrix was used to determine the optimal number of clusters. Estimation of Stromal and Immune cells in Malignant Tumor tissues using Expression (ESTIMATE) data were then employed to assess the stromal score and immune score of each OSCC sample. The Kaplan-Meier survival curve was performed for different TIME clusters using survminer R package.

### Differential Expression Analysis Based on TIME Phenotypes

OSCC patients were stratified into three TIME clusters on the basis of tumor immune cell infiltration. To filter the differentially expressed genes (DEGs) among different TIME subtypes, the genes related to TIME clusters were identified using the limma R package (Brorson et al., 2020). The threshold values of adjusted *p*-value < 0.05 and |log2 (fold change) | >1 were applied.

### Dimensionality Reduction and Measurement of TIME Score

First, consensus clustering was used to categorize the OSCC patients into different TIME gene clusters based on the expression of all DEGs by ConsensuClusterPlus R package. Then, the prognostic value of ferroptosis-related genes was computed by K-M analyses. *p*-value < 0.05 was deemed as statistically significant. Next, Boruta algorithm was employed to evaluate the dimensionality reduction of the distinct TIME gene signatures, and principal component 1 was extracted as the signature score via principal component analysis. Finally, a gene expression grade index was applied to measure the TIME score of each specimen:

TIMEscore=∑PC1x−∑PC1y

### Acquisition of Somatic Mutation Data

The genetic alteration data of OCSC patients were derived from the TCGA data portal (https://www.cancer.gov/tcga/) and applied as the training dataset. We identified the OSCC driver genes by maftools in R software and calculated the sum of somatic alterations in OSCC driver genes of each sample as the TMB score (Mayakonda et al., 2018). We also estimated the correlation between the TIME scores and the TMB scores (Zhang L et al., 2020). Next, the Kaplan-Meier survival curve for overall survival (OS) was constructed based on the high and low TMB score patterns using survminer R package. Another Kaplan-Meier survival curve was generated among the four different groups (high TMB score and high TIME score; high TMB score and low TIME score; low TMB score and high TIME score; low TMB score and low TIME score).

### Estimation of the Correlation Between Immune Cell Level and Gene Expression

TIMER is a novel algorithm used for characterizing the cell composition of a complex tissue and to estimate the level of tumor-infiltrating immune cells according to the corresponding gene expression profiles (Li et al., 2020). In this study, TIMER method was used to predict the tumor purity and immune score of each OSCC specimen from two TIME score clusters and to verify the correlation between the tumor-infiltrating immune cell level and the immune checkpoint gene expression level (CTLA4, PD-L1 and PD-1) estimated by CIBERSORT method (Wang et al., 2020).

### Gene Set Enrichment Analysis

GSEA database (http://software.broadinstitute.org/gsea/index.jsp) was employed to assess the difference in potential biological processes between high-risk and low-risk groups (*p* < 0.05).

### Statistical Analysis

All statistical tests were bilateral, and were carried out using R version 3.6.1 and Bioconductor (https://www.bioconductor.org/). The levels of significance (*p*-value) were set as <0.05, <0.01 < 0.001 and <0.0001.

## Results

### The Landscape of TIME-Infiltrating Cells in OSCC

**FIGURE 1:**
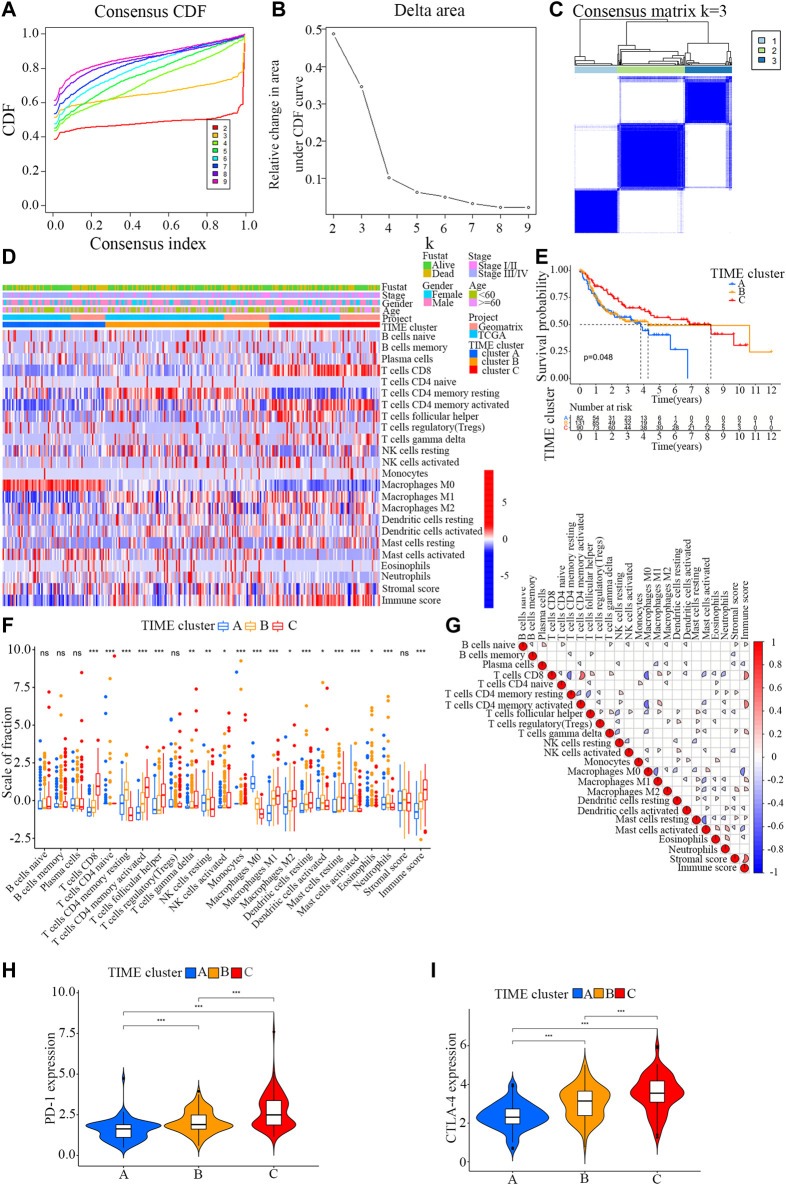
Landscape of the TIME-infiltrating cells of OSCC patients. **(A)** Cumulative distribution function curve of each consensus matrix from k = 2 to k = 9. **(B)** Relative changes in the area under CDF curve based on different k values. **(C)** Consensus clustering of the 304 tissue specimens derived from TCGA and GEO datasets for k = 3. **(D)** Unsupervised hierarchical clustering analysis of the TIME-infiltrating cells derived from TCGA and GEO datasets. Columns denotes tissue specimens and rows denotes TIME-infiltrating cells. **(E)** Overall survival of OSCC patients in the clusters **(A,B and C)**. Kaplan-Meier curves of OSCC patients with different TIME-infiltrating cell populations. Log-rank test, *p* = 0.048. **(F)** Distribution of the tumor-infiltrating immune cells in the clusters **(A,B and C)**. Statistical differences among the three TIME clusters were assessed by Kruskal-Wallis test. * *p* < 0.05; ** *p* < 0.01; *** *p* < 0.001. **(G)** Cellular interaction of the tumor-infiltrating immune cells among the three TIME clusters. **(H)** Difference in PD-1 expression among the three TIME clusters (Kruskal-Wallis test, *p* < 0.001). **(I)** Difference in CTLA-4 expression among the three TIME clusters (Kruskal-Wallis test, *p* < 0.001).

Firstly, the enrichment levels of TIME-infiltrating cells in OSCC tumor tissues were quantify using the CIBERSORT and ESTIMATE algorithms (Schelker et al., 2017; Yoshihara et al., 2013). Unsupervised clustering of 304 tumor samples from the meta-cohort (GSE41613 and tumor genome map [TCGA]) matched with tumor immune microenvironment (TIME) was carried out using the ConsesusClusterPlus package in R software, and the OSCC patients were divided into different subtypes through consensus clustering (Figures 1A–C; Supplementary File S3). (Gan et al., 2018) To compare the immune cell compositions of the TIME, we performed a heatmap for the three clusters (Figure 1D). Among the three distinct subtypes of TIME cell infiltration, the TIME cluster A exhibited a high level of M0 macrophage infiltration; the TIME cluster B demonstrated a significant increase in the amount of resting memory CD4^+^ T cells; and the TIME cluster C showed significant increases in the infiltration levels of CD8^+^ T cells, Follicular helper T cells and activated memory CD4^+^ T cells. The clinical characteristics of the three TIME clusters are presented in Table 1. Notably, OSCC patients in cluster C had a longer survival time (Log-rank test/Log rank, *p* = 0.048) than those in cluster A and B (Figure 1E), suggesting that TIME is associated with the prognosis of diffuse OSCC patients. Subsequently, the correlation coefficient heatmap was constructed, and the findings revealed that the infiltration of CD8^+^ T cells was positively correlated with the infiltration of CD4^+^ T cells (Figure 1G). Furthermore, we also analyzed the expression levels of the crucial immune checkpoints PD-1 and CTLA-4 in each TIME subtype (Goltz et al., 2018; Gabrych et al., 2019). The patients in TIME cluster C had significantly higher expression levels of CTLA-4 and PD-1 compared to cluster A and B.

**TABLE 1:** Characteristics of patients in cluster A, B, and C.

Characteristics	N	Cluster A	Cluster B	Cluster C
Total cases	304	82	131	91
Age				
<60	150	45	69	36
≥60	154	37	62	54
NA	1	0	0	1
Gender				
Male	210	61	88	61
Female	94	21	43	30
Stage				
I/II	81	18	30	33
III/IV	223	59	93	51
NA	20	5	8	7

### The Identified Immune Gene Subtype

**FIGURE 2:**
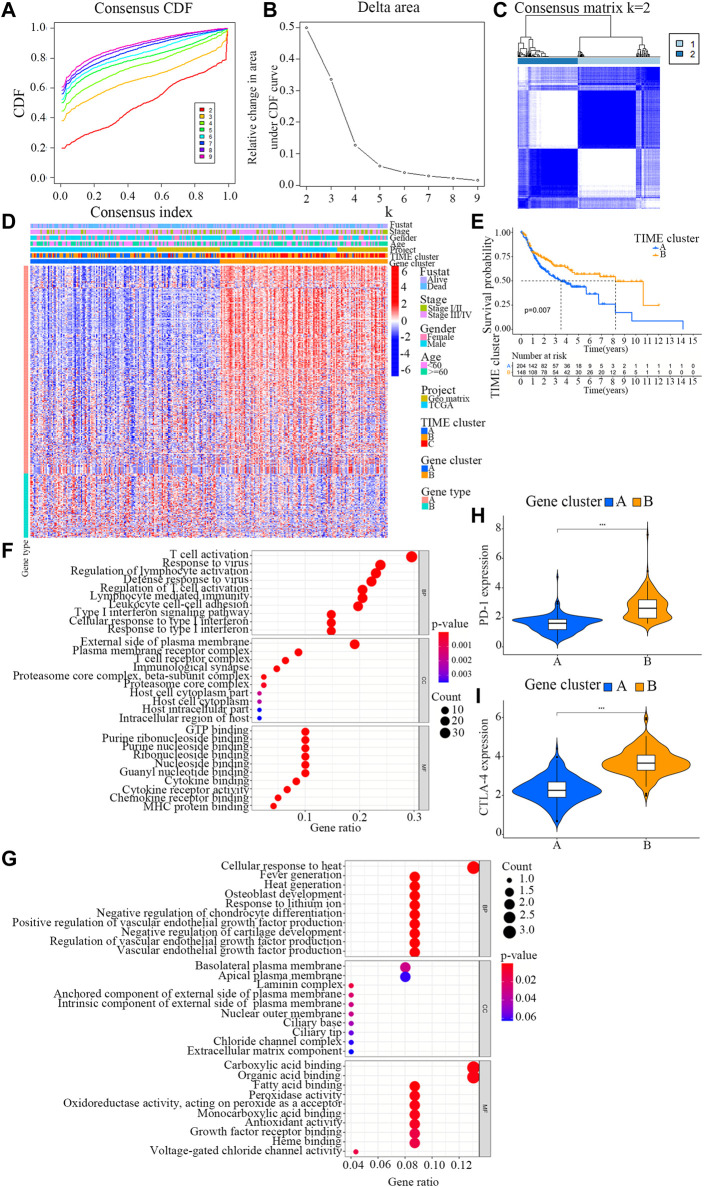
Identification of immunogenic gene subtypes. **(A)** Cumulative distribution function curve of each consensus matrix from k = 2 to k = 9. **(B)** Relative changes in the area under CDF curve based on different k values. **(C)** Consensus clustering of the 304 specimens derived from TCGA and GEO datasets for k = 2. **(D)** Unsupervised analysis clustering of common DEGs among three TIME clusters for classifying OSCC patients into Gene clusters **(A and B)**. **(E)** Overall survival of OSCC patients in the clusters **(A and B)**. Log-rank test, *p* = 0.007. **(F)** Bubble plot for the functional enrichment analyses of gene cluster **(A)**. The number of genes annotated to a GO term is indicated by the x axis. **(G)** Bubble plot for the functional enrichment analyses of gene cluster **(B)**. The number of genes annotated to a GO term is indicated by the x axis. **(H)** The distribution patterns of TIME-infiltrating cells in the two gene clusters. The immune scores of the two gene clusters are also plotted. * *p* < 0.05; ** *p* < 0.01; *** *p* < 0.001. **(I)** Difference in the expression levels of PD-1 and CTLA-4 between the two TIME gene clusters.

To explore differentially expressed genes among distinct immunophenotypes, limma R package was employed to compare the differences in mRNA expression levels among different subtypes. As a result, a total of 360 DEGs were identified in all OSCC samples. Then, an unsupervised clustering analysis was conducted on these 360 DEGs (Supplementary Files S4, S5), which classified the samples into two genome clusters, namely, gene clusters A and B (Figures 2A–C; Supplementary File S6). The 360 gene signatures were positively related to the gene cluster (TIME gene signature A), and the remaining were designated as TIME gene signature B (Supplementary File S7) (Kaymak et al., 2021). The expression of these DEGs is visualized in a heatmap (Figure 2D) by using the clusterProfiler R package (Zeng et al., 2019).

To further evaluate the prognosis of OSCC patients with different TIME gene clusters, the survival analysis in R programming language with a threshold of log-rank<0.05. It was observed that the patients in gene cluster B exhibited a better prognosis, whereas those in gene cluster A had a poor outcome (log rank test, *p* = 0.007; Figure 2E).

GO function analysis was carried out for TIME signature genes A and B respectively (Figures 2F,G; Supplementary Files S8, S9). Interestingly, the results showed that gene cluster B was markedly associated with higher immune scores (Figure 2H). The immune scores in the two TIME gene clusters were in accordance with the distribution patterns of TIME-infiltrating cells. The gene cluster A displayed a remarkable increase in the filtration of M0 macrophages, resting mast cells and neutrophils, while the gene cluster B exhibited a higher infiltration of activated CD4^+^ memory T cells, CD8^+^ T cells and M1 macrophages.

Additionally, the two TIME signature gene clusters demonstrated remarkable difference in the expression levels of PD-1 and CTLA-4 (Figure 2I). The PD-1/CTLA-4 expression level of TIME gene cluster B was relatively high, while that of TIME gene cluster A was slightly lower.

### Construction and Analysis of the TIME Score Groups

Principal component analysis was conducted to measure the TIME score of each sample, that is, the sum of the TIME score A and from TIME signature gene A and B, respectively, in order to obtain the quantitative index of TIME landscape (Supplementary File S10). The patients were stratified into high and low TIME score groups according to the optimal cut-off values obtained from survminer R package.

**FIGURE 3:**
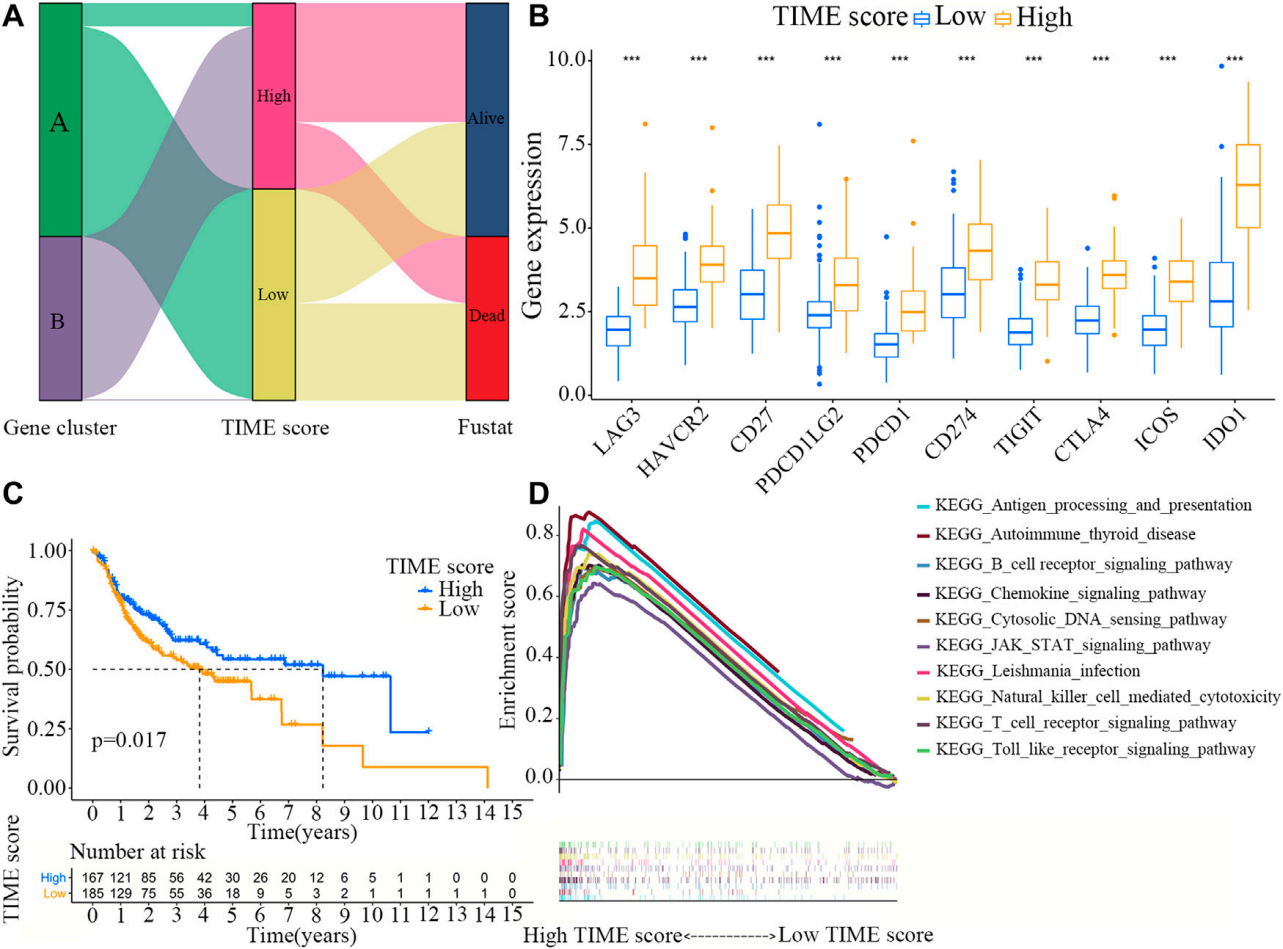
Construction and analysis of the TIME score groups. **(A)** Distribution patterns of OSCC patients in the two TIME gene clusters. **(B)** Expression levels of the immune checkpoint-related genes (LAG3, HAVCR2, CD27, PDCD1LG2, PDCD1, CD274, TIGIT, CTLA4, ICOS and IDO1) in high and low TIME score groups. **(C)** Survival rates of OSCC patients in high and low TIME score groups. Log-rank test, *p* = 0.017. **(D)** GSEA analyses of the genes in high TIME score group.

Figure 3A shows the distribution patterns of OSCC patients in the two gene clusters. The prognostic implication of TIME scores was evaluated, and the immune tolerance conditions of each group in the TCGA cohort were assessed before analyzing the prognostic values of TIME score in the TCGA cohort and other independent datasets. The immune checkpoint-related gene signatures of LAG3, HAVCR2, CD27, PDCD1LG2, PDCD1, CD274, TIGIT, CTLA4, ICOS and IDO1 were selected (Song et al., 2021). It was found that all these immune checkpoint-related genes were markedly overexpressed in high TIME score group, as revealed by the Wilcoxon test (Figure 3B). TIME score subtype analysis in the Kaplan-Meier plotter indicated that OSCC patients in high TIME score group exhibited markedly better survival rate than those in low TIME score group (log rank test, *p* = 0.017; Figure 3C). In addition, the results of GSEA demonstrated that the signaling pathways of B cell receptor, nature killer cell-mediated cytotoxicity, and toll-like receptor were significantly enriched in high TIME score group (Figure 3D).

### Correlation Between TIME Scores and Tumor Burden Mutation

**FIGURE 4:**
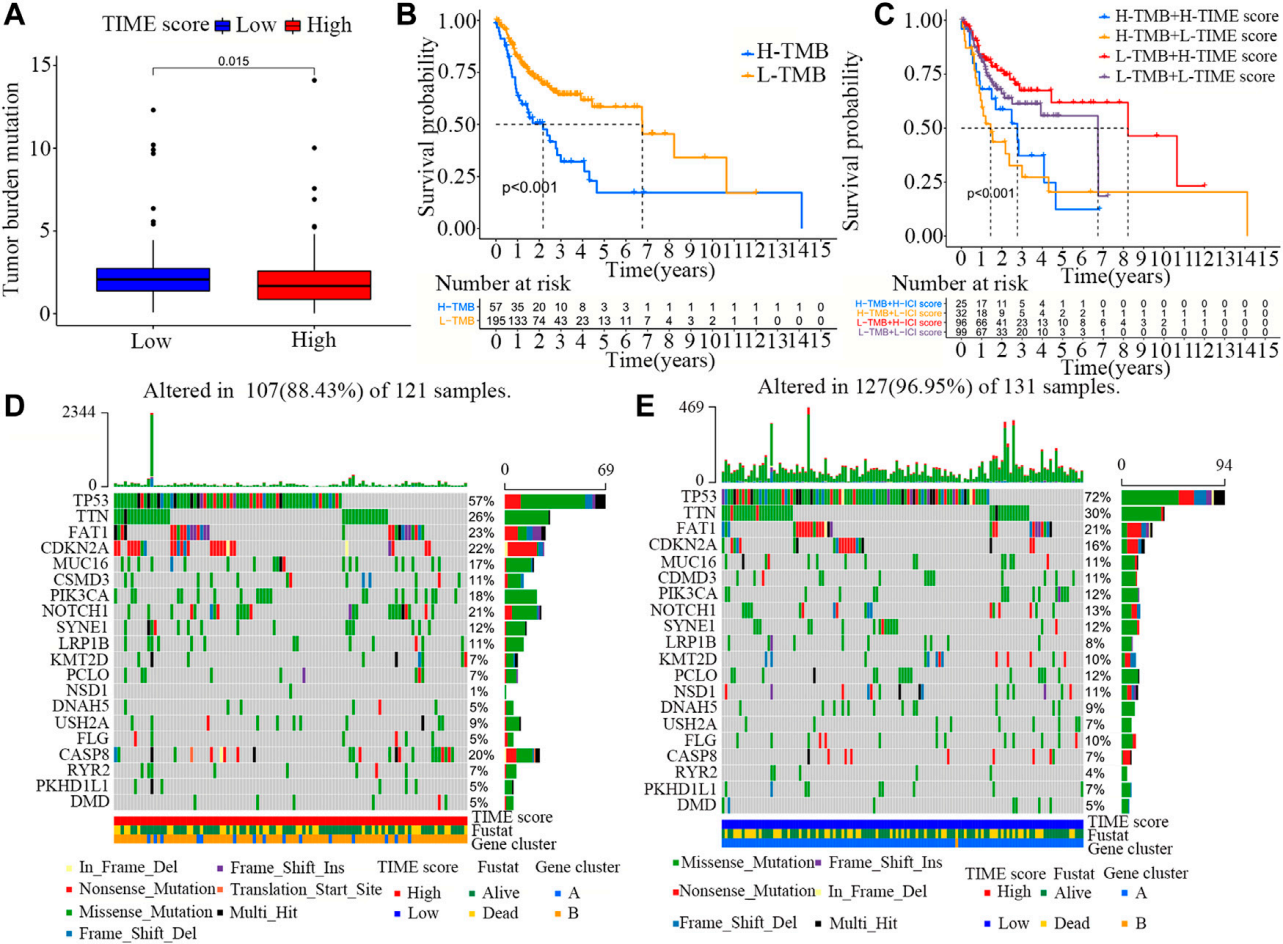
Association between TIME scores and somatic mutations. **(A)** Difference in TMB scores between high and low TIME score groups. Wilcoxon test, *p* = 0.015. **(B)** Survival rates of OSCC patients in high and low TMB score groups. Log-rank test, *p* < 0.001. **(C)** Survival rates of OSCC patients stratified by TIME and TMB scores. Log-rank test, *p* < 0.001. **(D)** Construction of the oncoPrint based on high TIME scores on the left (red). **(E)** Construction of the oncoPrint based on low TIME scores on the right (blue).

TMB is an important molecular marker for assessing the outcomes of tumor immunotherapy (Mayakonda et al., 2018). Previous evidence shows that the level of TMB can not only predict the efficacy of immunotherapy, but also accurately predict the efficacy of many targeted and chemotherapeutic drugs (Zhu et al., 2019b). Besides, TMB also has important clinical significance. In this study, we attempted to determine the relationship between TMB and TIME scores. First, the TMBs of OSCC patients with high and low TIME scores were compared (Supplementary File S11). As shown in Figure 4A, the TMB of OSCC patients was higher in low TIME score group than in high TIME score group (Wilcoxon test, *p* = 0.015). Next, we categorized the patients into high and low TMB groups. As shown in Figure 4B, OSCC patients in low TMB group exhibited better survival rate than those in high TMB group (log-rank test, *p* < 0.001). Based on these contradictory results, we further explored the combined effect of TMB and TIME scores on OSCC prognosis. Stratified survival analysis showed that TMB scores did not affect TIME scores-based prognostic prediction. However, a significant difference in survival rates was observed between high and low TMB groups stratified by TIME scores (Figure 4C).

In addition, the distribution patterns of somatic alterations in OSCC patients were compared between high and low TIME groups. The R package maftools was used to identify potential driver genes in OSCC patients. The top 20 variant mutated driver genes were selected for further analysis. The annotated mutation files were downloaded from TCGA database and then analyzed. As shown in Figures 4D,E and Table2, the frequencies of NSD1, CASP8 and TP53 mutations were noticeably different between high and low TIME score groups. These results may provide new insights into the mechanisms of gene mutations and TIME compositions as well as novel targets for immunotherapy.

**TABLE 2:** Association of TIME scores with somatic variants.

Gene symbol	High ICI score (%)	Low ICI score (%)	p-value
TP53	68 (57)	94 (72)	0.0144
TTN	31 (26)	39 (30)	0.4643
FAT1	27 (23)	27 (21)	0.7374
CDKN2A	26 (22)	20 (16)	0.2059
MUC16	20 (17)	14 (11)	0.2459
CSMD3	13 (11)	14 (11)	0.9884
PIK3CA	21 (18)	15 (12)	0.1874
NOTCH1	25 (21)	17 (13)	0.1028
SYNE1	14 (12)	15 (12)	0.9649
LRP1B	13 (11)	10 (8)	0.3937
KMT2D	8 (7)	13 (10)	0.4869
PCLO	8 (7)	15 (12)	0.2067
NSD1	1 (1)	14 (11)	0.0005
DNAH5	6 (5)	11 (9)	0.1972
USH2A	10 (9)	9 (7)	0.5166
FLG	6 (5)	13 (10)	0.1369
CASP8	24 (20)	9 (7)	0.0022
RYR2	8 (7)	5 (4)	0.3182
PKHD1L1	6 (5)	9 (7)	0.5236
DMD	6 (5)	6 (5)	0.8908

TIME-tumor immune microenvironment; p value was obtained from the chi-square test between different TIME score subtypes.

### Prognosis Analysis Between High and Low TIME Score Groups With Specific Clinical Characteristics

**FIGURE 5:**
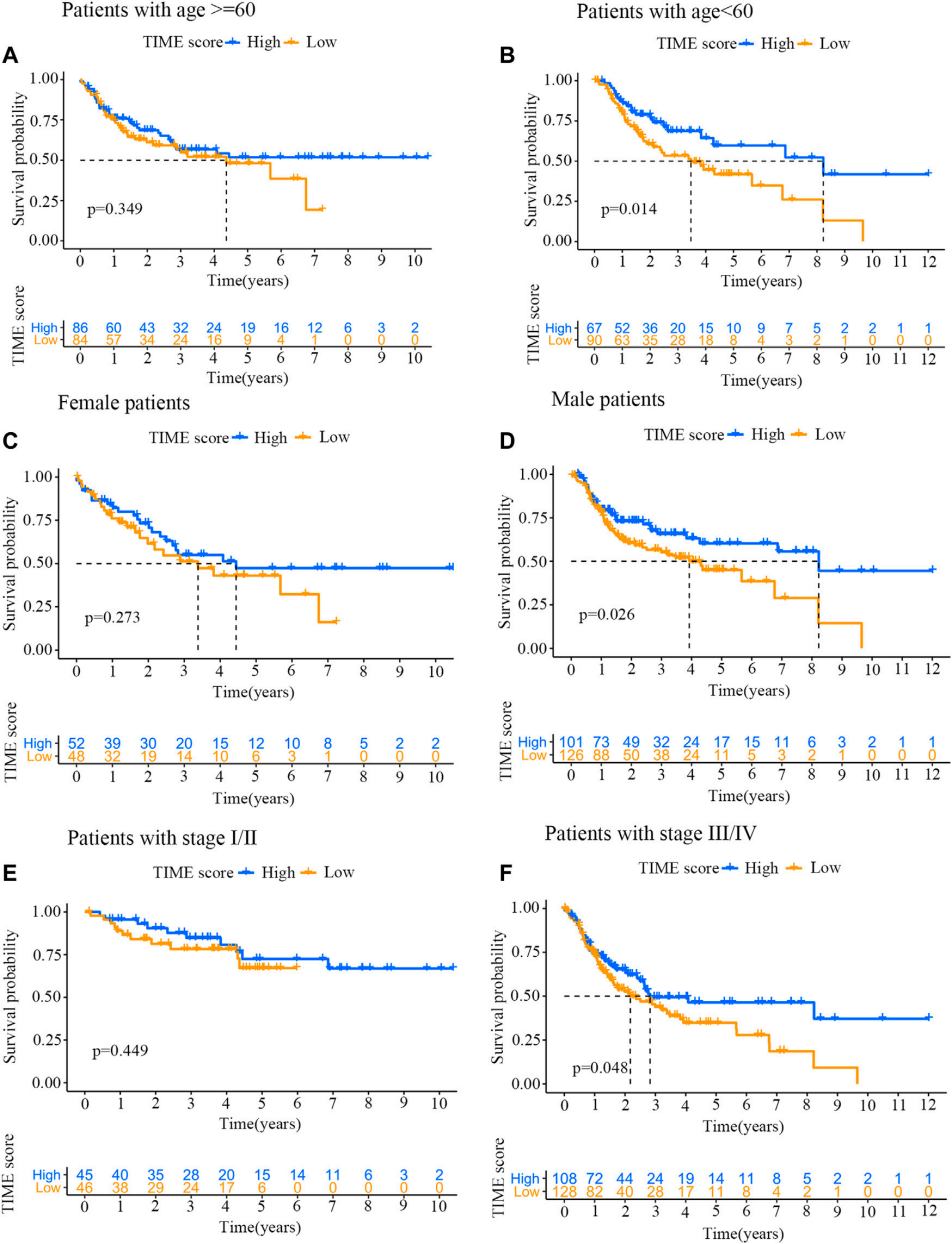
Prognosis analysis of high and low TIME score groups with specific clinical characteristics. **(A)** Survival curves for patients (age ≥60 years old) from high and low TIME score groups. Log-rank test, *p* = 0.349. **(B)** Survival curves for patients (age <60 years old) from high and low TIME score groups. Log-rank test, *p* = 0.014. **(C)** Survival curves for female patients from high and low TIME score groups. Log-rank test, *p* = 0.273. **(D)** Survival curves for male patients from high and low TIME score groups. Log-rank test, *p* = 0.026. **(E)** Survival curves for stage I/II patients from high and low TIME score groups. Log-rank test, *p* = 0.449. **(F)** Survival curves for stage III/IV patients from high and low TIME score groups. Log-rank test, *p* = 0.048.

To further evaluate the prognostic implication of TIME scores on OSCC patients, all samples were categorized into different groups by clinical characteristics (e.g., gender, stage and age). The survival curves of TIME scores were constructed using R software with survival package. As shown in Figures 5A,B, TIME scores were positively correlated with OS among patients younger than 60. However, no marked difference was found between high and low score groups with age over 60. The TIME scores were also positively correlated with OS among male patients (Figures 5C,D) and stage Ⅲ/Ⅳ patients (Figures 5E,F).

## Discussion

Previous research has shown that immune system is involved in the occurrence and metastasis of OSCC patients (Abbott and Ustoyev, 2019). TIME cells play a double-edged sword role, which not only recognize tumor cells and inhibit tumor development, but also help tumor cells achieve immune escape and promote tumor development (Lakshmi Narendra et al., 2013; Dogan et al., 2018). As the immune status is closely related to OSCC, the new immunotherapy of OSCC has received widespread attention. The main purpose of tumor immunotherapy is to activate the damaged immune system by rescuing failed T cells and regulating immunosuppressive cells. In 2016, PD-1 targeting drugs (Pembrolizumab) and anti-PD-1 monoclonal antibody (nivolumab) were approved by the US Food and Drug Administration (FDA) for treating recurrent or metastatic OSCC patients (Kujan et al., 2020; Tang et al., 2020). Previous studies have confirmed the relative safety of anti-PD-1/PD-L1 agents, but the primary drug resistance rate to PD-1/PD-L1 is as high as 60% in patients with malignant tumors such as OSCC (Topalian et al., 2012). Considering that this immunotherapy may not benefit all OSCC patients, we quantified the TIME scores of OSCC tumor and used this scoring system to evaluate the prognostic biomarkers of immunotherapy response.

Specifically, the TIME scores of 257 OSCC samples were analyzed and subsequently used to classify OSCC patients into three different immune subtypes. In the analysis, we found that the concentrations of CD4+T, CD8+T and helper T cells were associated with higher TIME scores, and these patients showed a good prognosis. Previous studies have shown that hot tumors reflect good immunogenicity, and CD4+T and CD8+T cells continue to infiltrate into tumor stroma, para-tumoral areas and tumor nests (Ochoa de Olza et al., 2020). These subtypes respond well to TIME therapy such as PD-1 in clinic (Lenouvel et al., 2020). We also found that immune checkpoints, such as PD-1 and CLAT4, showed high expression levels in groups with high immune scores. According to the new classification of gene clusters, it was observed that the gene cluster B showed relatively high immune score, inflammatory cell density and a more favorable activation phenotype, with the highest densities of CD4+T cells, CD8+T cells and M1 macrophages. On the other hand, the gene cluster A had a lower TIME score and displayed an immune cold phenotype, and the patients exhibited a poor prognosis (Ochoa de Olza et al., 2020). Therefore, it is speculated that OSCC patients in TIME gene cluster B may actually benefit from immunotherapy. Nevertheless, due to the heterogeneity of TIME scores, tumor subtype-specific biomarkers should be established for improving OSCC prognosis prediction.

Through GSEA pathway analysis, we found that toll-like receptor and B cell receptor signaling pathways were remarkably enriched in high TIME score group. Previous research has found that the increased expression of B cell receptor signaling pathways Fos and Jun as the direct downstream of the activated MAPK pathway and the end point of this pathway can produce an immune response (Zhao et al., 2009; Das et al., 2019). The sensitivity of TLR2 could be increased after the malignant phenotype of OSCC was developed. Hence, this gives us a new choice when seeking for new targets for immunotherapy. Additionally, it can be seen from the survival analysis of OSCC patients that TMB is not related to TIME, and thus can be used as an independent indicator. By comparing the difference between TIME high and low score groups, the three genes with the highest mutation frequency were identified, which could serve as good candidates for predicting the responses to immunotherapy. In addition, younger OSCC patients (less than 60 years old) with high TIME scores showed better survival rates, and OSCC stage III/IV patients also exhibited the same results. Previous studies have shown that male patients do not appear to benefit from immunotherapy compared with female patients (Lin et al., 2019; Yang F et al., 2020). However, our study revealed that male patients with high TIME scores had remarkably higher survival rates than female patients. This may be helpful to our search for immune targeted cancer therapy in the future.

In summary, we obtained potential “subtype biomarkers” through the Boruta algorithm and established a TIME scoring system to describe the TIME characteristics of OSCC patients in a relatively comprehensive way and gain a deeper understanding of tumor immune infiltration (Peng et al., 2021). By comparing the TIME scores, we proved the feasibility of this scoring system for assessing the responses of targeted immunotherapy. In the near future, we should apply the TIME score model into clinical practice, in order to improve the accuracy of OSCC diagnosis and provide more high-quality programs for patients’ immunotherapy.

## Conclusion

Overall, we used two algorithms to reveal the characteristics of immune microenvironment of oral squamous cell carcinoma. The analysis shows that the immune microenvironment score can be used as an independent index of treatment and prognosis. This study provides a new perspective for immunotherapy of oral squamous cell carcinoma and provides a new strategy for immunotherapy in the future.

## Data Availability

The original contributions presented in the study are included in the article/Supplementary Material, further inquiries can be directed to the corresponding author.

## Abbreviations

AUC, area under the curve; CDF, Cumulative Distribution Function; CTLA4, cytotoxic T lymphocyte antigen 4; DEG, differentially expressed gene; ESTIMATE, Estimation of Stromal and Immune cells in Malignant Tumor tissues using Expression data; GEO, Gene Expression Omnibus database; GO, Gene Ontology; GSEA, gene set enrichment analysis; K-M, Kaplan-Meier; OS, overall survival; OSCC, oral squamous cell carcinoma; PD, programmed death 1; PDL1, programmed cell death-ligand 1; ROC, receiver operating characteristic; TCGA, The Cancer Genome Atlas; TIME, tumor immune microenvironment; TMB, Tumor Mutation Burden.
